# Efferocytosis of apoptotic alveolar epithelial cells is sufficient to initiate lung fibrosis

**DOI:** 10.1038/s41419-018-1074-z

**Published:** 2018-10-17

**Authors:** Kevin K. Kim, Megan R. Dotson, Manisha Agarwal, Jibing Yang, Patrick B. Bradley, Natalia Subbotina, John J. Osterholzer, Thomas H. Sisson

**Affiliations:** Department of Internal Medicine, Division of Pulmonary and Critical Care Medicine, University of Michigan, Ann Arbor, MI 48109 USA

## Abstract

Type II alveolar epithelial cell (AEC) apoptosis is a prominent feature of fibrotic lung diseases and animal models of pulmonary fibrosis. While there is growing recognition of the importance of AEC injury and apoptosis as a causal factor in fibrosis, the underlying mechanisms that link these processes remain unknown. We have previously shown that targeting the type II alveolar epithelium for injury by repetitively administering diphtheria toxin to transgenic mice expressing the diphtheria toxin receptor off of the surfactant protein C promoter (SPC-DTR) develop lung fibrosis, confirming that AEC injury is sufficient to cause fibrosis. In the present study, we find that SPC-DTR mice develop increased activation of caspase 3/7 after initiation of diphtheria toxin treatment consistent with apoptosis within AECs. We also find evidence of efferocytosis, the uptake of apoptotic cells, by alveolar macrophages in this model. To determine the importance of efferocytosis in lung fibrosis, we treated cultured alveolar macrophages with apoptotic type II AECs and found that the uptake induced pro-fibrotic gene expression. We also found that the repetitive intrapulmonary administration of apoptotic type II AEC or MLE-12 cells induces lung fibrosis. Finally, mice lacking a key efferocytosis receptor, CD36, developed attenuated fibrosis in response to apoptotic MLE-12 cells. Collectively, these studies support a novel mechanism linking AEC apoptosis with macrophage pro-fibrotic activation via efferocytosis and reveal previously unrecognized therapeutic targets.

## Introduction

Progressive alveolar fibrosis is a serious complication of certain systemic inflammatory disorders, inorganic and organic dust exposures, drug toxicity and primary diseases of the lung including idiopathic pulmonary fibrosis (IPF)^1–5^. Mounting evidence implicates defects in the type II alveolar epithelial cell (AEC) in disease pathogenesis^6^. For example, histopathologic abnormalities of the epithelium including apoptosis are observed in tissue sections from IPF patients and in animal models of pulmonary fibrosis^7–9^. Furthermore, mutations in type II AEC genes including surfactant proteins A and C are linked to familial disease^10^. Finally, transgenic animal experiments from our laboratory confirm that targeted injury to the type II alveolar epithelium is sufficient to initiate lung scarring^11^.

Despite the substantial evidence linking type II AEC injury/death to the development of fibrosis, the pathways that translate an epithelial insult into lung collagen accumulation have not been well-characterized. Possible mechanisms by which damage to the alveolar epithelium lead to fibrosis have focused on either loss of anti-fibrotic functions supplied by healthy cells or an up-regulation of pro-fibrotic factors from the injured AECs. An alternative mechanism supported by emerging evidence suggests that the apoptotic AECs can directly trigger progressive fibrosis by inducing a response in neighboring cells.

Cellular apoptosis terminates with fragmentation resulting in formation of vesicles termed apoptotic bodies. Apoptotic bodies are characterized in part by the appearance of phosphatidylserine on the outer leaflet of the lipid bilayer which serves as a recognition signal for phagocytic cells to ingest the cellular debris. Apoptotic cells and bodies modulate cell behavior as they undergo phagocytosis in a process known as efferocytosis^12^. For example, in models of acute lung injury, efferocytosis of apoptotic neutrophils has emerged as a key pathway in regulating macrophage function and restoring homeostasis by promoting release of anti-inflammatory cytokines^13^. The ingestion of apoptotic neutrophils is well studied and involves protein receptors expressed on the surface of the ingesting cells and the apoptotic bodies^12^. Of note, there is considerable overlap between anti-inflammatory and pro-fibrotic pathways as exemplified by one report that found the anti-inflammatory effects of macrophages which had ingested apoptotic cells resulted from the increased expression of TGFβ1 (a well-established pro-fibrotic cytokine)^13,14^. Further evidence linking apoptotic cells with lung fibrosis comes from a report in which the administration of a single dose of lavaged alveolar cells (presumably macrophages) induced to undergo apoptosis caused a fibrotic response in mice^15^. Although much less is known about the fate of apoptotic AECs and whether their uptake by macrophages might be an important inciting event in fibrosis, we hypothesized that the efferocytosis of apoptotic type II AECs would significantly contribute to the initiation of fibrosis following lung injury. To test this hypothesis, we employed a transgenic model of fibrosis in which mice engineered to express the diphtheria toxin receptor (DTR) on their type II AECs are treated with repeated doses of diphtheria toxin (DT)^11^. We also directly administered repeated doses of apoptotic AECs into the lungs of healthy mice. We found that targeted epithelial injury led to apoptosis and evidence of macrophage ingestion of apoptotic cells. We also determined that the intrapulmonary administration of apoptotic AECs was sufficient to cause lung fibrosis through CD36-dependent efferocytosis. Together these findings indicate that the uptake of apoptotic type II AECs by lung macrophages in the setting of lung injury is a critical initiating step in fibrosis pathogenesis.

## Methods and materials

### Mice

All animals were housed in a specific pathogen-free environment until the day of sacrifice, and each in vivoll murine experiments was approved by the University of Michigan Animal Care and Use Committee. Wild-type and transgenic mice were on a C57Bl/6 background. SPC-DTR mice in which expression of the diphtheria toxin receptor is regulated by a modified surfactant protein-C promoter are previously described^11^. Control or SPC-DTR mice were treated with daily IP injections of DT at the timepoints indicated. CD36-null mice were purchased from Jackson Laboratories and bred in our animal facilities. Oropharyngeal delivery of apoptotic cells to mice was performed following the method of Lakotos et al. ^16^. For BAL collection, mice were sacrificed at the timepoints indicated and lungs lavaged with 1 mL of PBS.

### Reagents

AdGFP was purchased from University of Iowa Viral Vector Core. Matrigel, biotin-conjugated rat anti-mouse CD45 antibody, biotin-conjugated rate anti-mouse CD16/32 antibody, and PE-conjugated rat anti-mouse CD45 antibody were purchased from BD Biosciences. Streptavidin conjugated and magnetic separator are from ThermoFisher. Mouse/Rat/Porcine/Canine TGFβ ELISA kit was purchased from R&D Systems. Low melting point agarose is from Life Technologies. Small airway growth media (SAGM) is from Lonza. Keritinocyte growth factor (KGF) is from Peprotech. Pro-surfactant protein-C (pro-SPC) antibody is from EMD Millipore. All other reagents are from Sigma Pharmaceuticals.

### Hydroxyproline assay

Lung collagen content was determined by an assay for hydroxyproline as previously described^17,18^. Briefly, mice were sacrificed at the timpoints indicated. Lungs were removed, homogenized and incubated in 12 N HCl at 120 °C for 16 h. Aliquots of each sample were mixed with citrate buffer and chloramine T solution. Erlich’s solution was added and each sample was incubated for 15 min at 65 °C. Absorbance at 540 nm was measured and the hydroxyproline concentration was determined against a standard curve.

### Cell isolation and culture

Murine primary AECs were isolated and cultured as previously descibed^17,18^. Briefly, mice were sacrificed and lungs were injected with dispase followed by low melting point agarose. Lung were removed and digested in dispase. The crude dissected lungs were filtered to remove larger chunks and the resulting cells were negatively selected with anti-CD45 and anti-CD16/32 biotin-conjugated antibiodies using streptavidin-magnetic beads. The resulting AECs were further negatively selected by incubating on a sterile petri dish for 1–2 h at 37 °C. Primary AECs were cultured in SAGM supplemented with KGF on Matrigel-coated plates. In some experiments, cells were treated with AdGFP (50 pfu/cell) for one day. The cells were rinsed to remove any free adenovirus and were covered with fresh media for 24 h prior to induction of apoptosis. MLE-12 cells were purchased from ATCC and cultured in HITES media as previously described^11^. Jurkat cells were purchased from the ATCC and cultured in RPMI per ATCC protocol. Primary murine alveolar macrophages were isolated by serial bronchoalveolar lavage as previously described^19^. Breifly, mice were anesthetized and lungs were exposed. Lungs were sequentially lavaged 10 times with 1 ml of EDTA in PBS. The pooled lavage fluid was centrifuged at 100 x g and the cell pellet was resuspended and cultured in DMEM supplemented with 10% FBS. All cells were cultured in a 37 °C, 5% CO_2_, incubator.

### UV-induced apoptosis and Intrapulmonary delivery

For intrapulmonary delivery of apoptotic bodies derived from primary AEC, cultured primary AECs were subjected to 30 min of UV light (10.0 × 10^5^ microjoules) using a UV Stratralinker 1800 (Stratagene). UV treated cells were placed back in the tissue culture incubator for 24 h to ensure sufficient time for apoptotic body generation. Conditioned media containing apoptotic cells was then collected and centrifuged at 1200 x g to isolate the apoptotic cells. The pellet was resuspended in 50 µl of PBS for delivery to mice. Live AECs (5 × 10^5^) or apoptotic AECs (5 × 10^5^) or PBS control were delivered to mice by oropharyngeal aspiration as previously described^16,20^ to allow for repetitive dosing. Mice were treated with daily doses for seven days. Mice were sacrificed 21 days after the initial dose for assessment of lung fibrosis.

For intrapulmonary delivery of apoptotic MLE-12 cells conditions were optimized to yield higher levels of fibrosis. MLE-12 cells were grown in 100 × 20 mm cell culture dishes to a confluency of 100%. Media was removed, cells were washed and incubated in 500 µl of PBS prior to exposure with ultraviolet-C (254 nm) radiation for a total of 10.0 × 10^5^ microjoules. The cell culture dish was rotated 90 degress and exposed again. The cultures were incubated for 1 h at 37 °C and scraped. Cells were counted with a sceptor hand held cell counter, and 5.0 × 10^5^ cells in 25–40 µl total volume were delivered to mice anesthetized with isoflurane by oropharyngeal aspiration. Apoptotic cell adminsitration was repeated on Monday, Wednesday, and Friday for three consecutive weeks, and lungs were harvested on the last day 4 h after the final instillation.

### TGFβ ELISA

Level of active TGFβ was determined from conditioned media or BAL samples using the murine/mouse/rat/porcine/canine TGFβ ELISA kit per manufacturers protocol and values quantified against a standard curve.

### Gene expression analysis

RNA was isolated from cells with TRIzol (Life Technologies) following manufacturer’s instructions. Reverse transcription was performed with the SuperScript III first-strand synthesis kit (Life Technologies) and RT-qPCR was performed using the POWER SYBR green PCR mastermix kit (Applied Biosystems) and Applied Biosystems 7000 sequence detection system. The relative expression levels of gene in fold changes were calculated against GAPDH. Primers sequences are GAPDH forward: 5’-AACTTTGGCATTGTGGAAGG-3’, GAPDH reverse: 5’-ACACATTGGGGGTAGGAACA-3’, TGFβ forward: 5’-ATCCTGTCCAAACTAAGGCTCG-3’, TGFβ reverse: 5’-ACCTCTTTAGCATAGTAGTCCGC-3’, arginase forward: 5’-CAGAAGAATGGAAGAGTCAG-3’, arginase reverse: 5’-CAGATATGCAGGGAGTCAC-3’, iNOS forward: 5’-CCCTTCAATGGTTGGTACATGG-3’ and iNOS reverse: 5’-ACATTGATCTCCGTGACAGCC-3’.

### Flow cytometry

Detection of efferocytosis by uptake of GFP-positive apoptotic lung epithelial cells by non-epithelial, CD45-positive cells was performed on BAL cells by flow cytometry using a modified previously reported protocol^21^. Briefly, cells were resuspended in blocking buffer containing 1% BSA in PBS for 30 min. Cells were then incubated in PE-conjugated rat anti-mouse CD45 antibody in blocking buffer for 30 min. Cells were washed and analyzed by flow cytometry using an attune flow cytometer (Life Technologies). Data was analyzed using FloJo (Treestar) software.

### Cell death assays

Surface expression of phosphatydylserine was determined using TACS Annexin V-FITC Apoptosis detection kit (R&D Systems) per manufacturer’s protocol. Terminal deoxynucleotidyl transferase dUTP-mediated nick-end labeling (TUNEL) was performed on lung sections using the in situ death detection kit and TMR red as previously described^9,20^.

### Statistical analysis

Data are expressed as mean values ± SEM. Student’s *t*-test (2-tailed) was used to determine differences for experiments between two groups and 1-way ANOVA was used to determine differences for experiments with more than two groups. *P* value of < 0.05 was accepted as statistically significant.

## Results

### Increased lung apoptosis following targeted type II alveolar epithelial cell injury

**Fig. 1:**
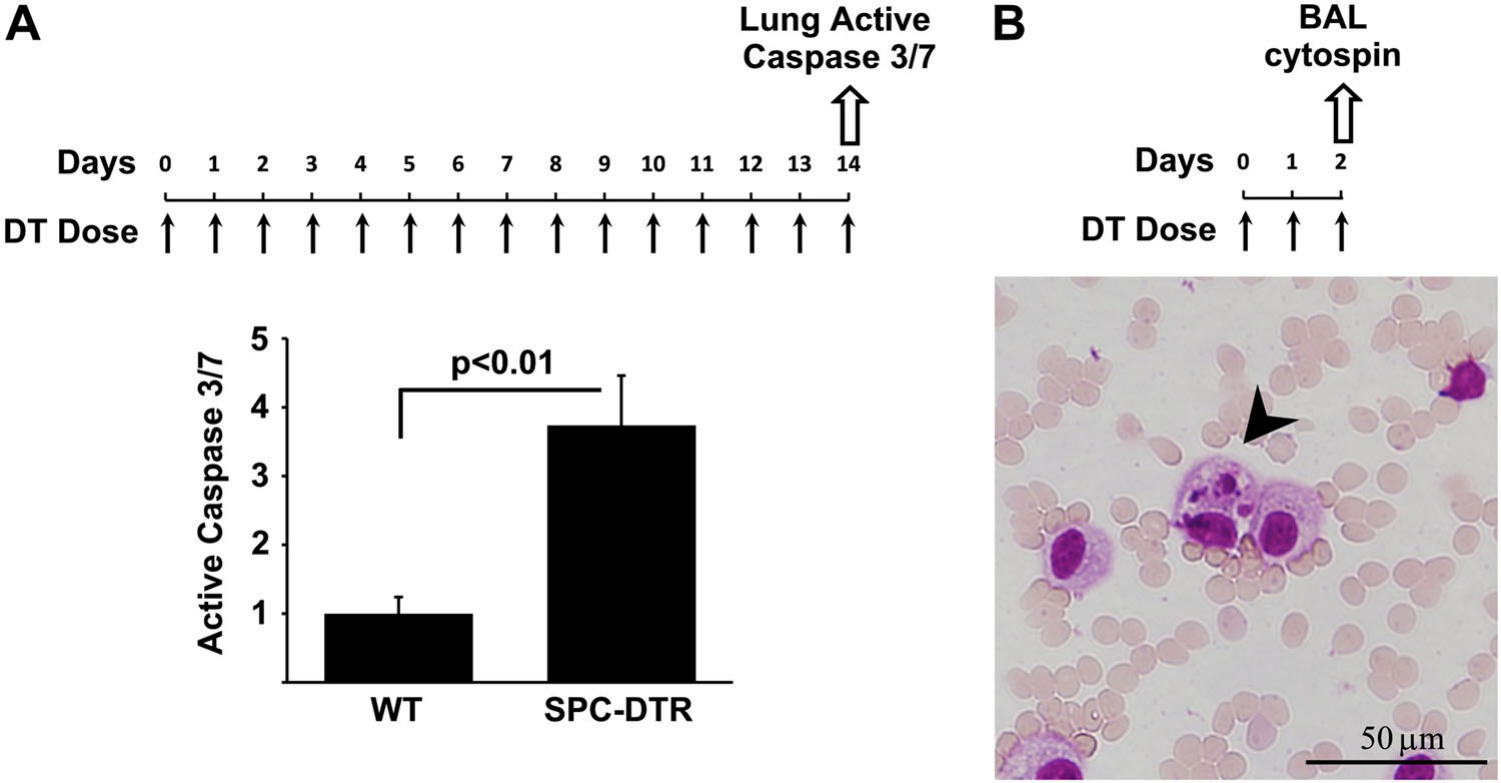
Increased lung apoptosis following targeted type II alveolar epithelial cell injury. **a** Transgenic SPC-DTR mice treated with daily doses of diphtheria toxin (DT, 10 µg/kg i.p.) for 14 days have increased levels of active caspase 3/7 compared to WT mice. *N* = 4, *p* < 0.01. **b** Two days after daily doses of diphtheria toxin (DT, 10 µg/kg i.p.) bronchoalveolar lavage cells were stained H&E (100x). A subset of alveolar macrophages contain apoptotic bodies (arrowhead)

We have previously described the generation of transgenic mice (SPC-DDTR) in which the murine surfactant promoter C promoter drives diphtheria toxin receptor expression in a type II AEC-specific manner^11^. Treatment of the SPC-DTR mice with repeated daily doses of DT for 14 days results in the devlopment of pulmonary fibrosis. In this model, the fate of the targeted type II AECs is unknown. To determine whether the DT-mediated injury is associated with increased caspase-mediated apoptosis, we harvested lungs from SPC-DTR mice two days after initiation of DT treatment. A control group of WT animals was treated with PBS. Caspase-mediated apoptosis was measured in whole lung lysates with an assay for active caspase 3/7. We found that DT treatment resulted in a marked increase in caspase 3/7 activity in SPC-DTR mice (Fig. 1a) and TUNEL-staining within pro-SPC-positve cells (Supplemental Fig. 1), confirming that the targeted insult leads to increased apoptotic cell death. Control mice exhibited significantly lower levels of active caspase 3/7 and TUNEL-positive cells. To assess for evidence of clearance of apoptotic AECs by alveolar macrophages following DT-mediated targeted injury, we performed cytospins of bronchoalveolar lavage fluid from SPC-DTR mice treated with DT for two days. We found in the lavage fluid the presence of apoptotic body-containing macrophages (Fig. 1b). Collectively, these results indicate that DT injury of SPC-DTR results in the induction of apoptosis in type II AECs with associated efferocytosis by alveolar macrophages.

### Macrophage ingestion of apoptotic alveolar epithelial cells

**Fig. 2:**
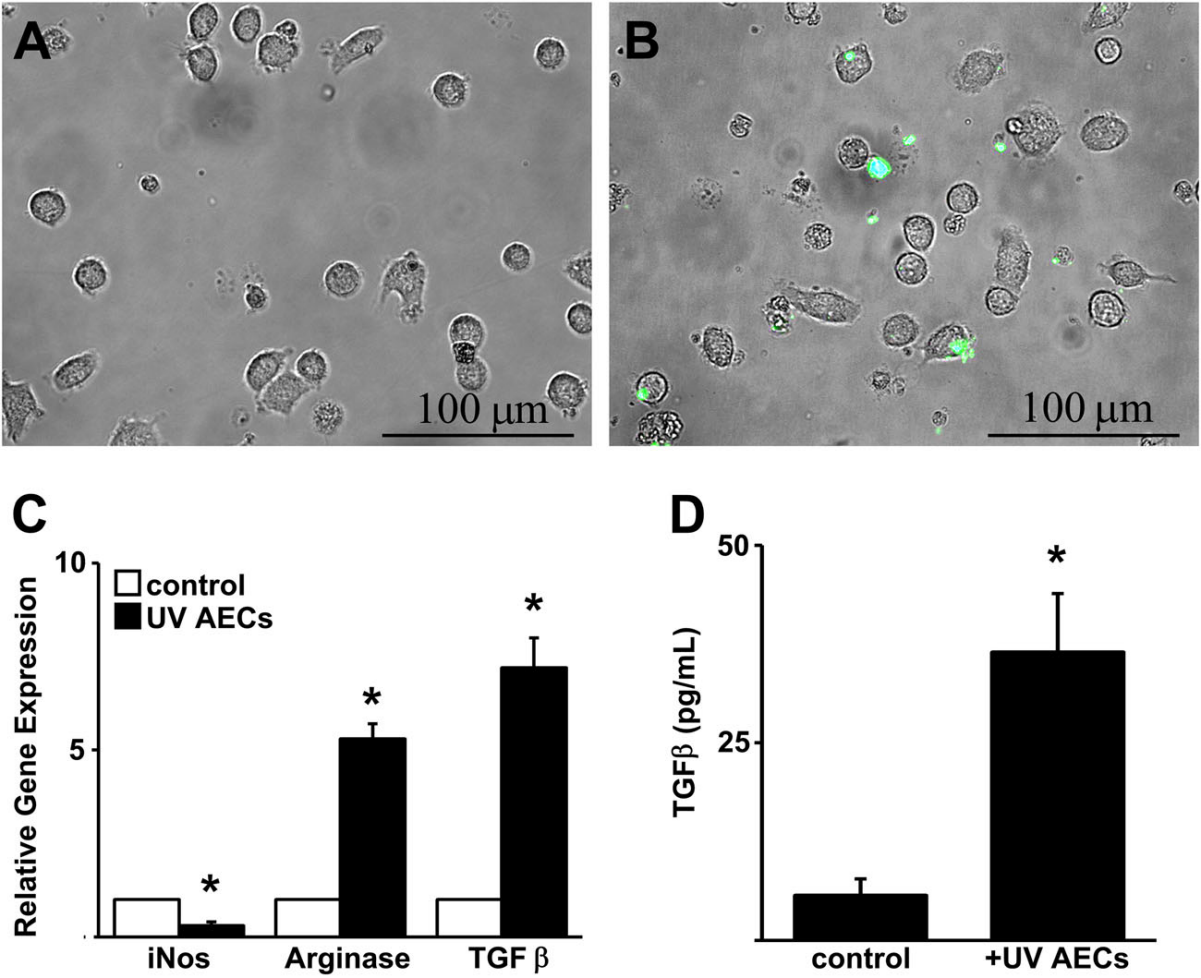
Alveolar macrophage efferocytosis of apoptotic type II alveolar epithelial cells induces an alternatively activated (M2) polarization **a**, **b** Overlay images (20×) of phase and fluorescent microscopy images of cultured alveolar macrophages treated under control conditions **(a)** or treated for 2 h with UV-exposed GFP-labeled primary alveolar epithelial cells **(b)**. **c** RT-qPCR expression analysis of cultured primary alveolar macrophages treated UV-treated primary AECs or without (control) for 24 h. **d** TGFβ ELISA of conditioned media from alveolar macrophages treated UV-treated primary AECs for 24 h. *N* = 4, *p < 0.05 compared to unstimulated macrophages

Prior studies demonstrate that the ingestion of apoptotic cells by macrophage leads to a phenotypic switch. To determine if the efferocytosis of apoptotic AECs is sufficent to induce a profibrotic phenotype in alveolar macrophages, we induced apoptosis in cultured GFP-labeled primary type II AECs via exposure to UV radiation. Surface expression of phosphatidylserine was confirmed by flow cytometry (Supplemental Fig. 2). We then adminsitered the GFP-labeled apoptotic type II AECs to primary alveolar macrophages for 2 h and washed the cultures to remove any free apoptotic cells. Fluorescent microscopy was then employed to assess for evidence of efferocytosis. We found that a subset of macrophages contained GFP-positive apoptotic cells consistent with efferocytosis (Fig. 2a, b). To determine if uptake of apoptotic type II AECs led to a phenotypic alteration in the ingesting macrophage, we performed an mRNA expression analysis 24 h after treatment. We found that macrophage exhibited increased expression of arginase and TGFβ and a reduction in the expression of iNOS compared to alveolar macrophages that were not exposed to apoptotic cells (Fig. 2c). Release of TGFβ into the conditioned media by the efferocytotic macrophages was confirmed by ELISA (Fig. 2d).

To determine if this macrophage response was unique to apoptotic AECs, we first compared the response of alveolar macrophages following exposure to UV-treated AECs versus UV-treated Jurkat cells. The administration of equal numbers of UV-treated AECs and Jurkat cells both induced an upregulation of arginase and TGFβ in the ingesting macrophage. However, the magniutude of response to UV-treated AECs was significantly greater (Supplemental Fig. 3A&B). Next we compared the gene expression response of alveolar macrophages folowing exposure to apoptotic versus live AECs. In order to avoid adherence of live AECs to the tissue culture plate, we cultured alveolar macrophages on non-tissue culture treated petri dishes. Macrophages were then administered AECs that had been UV-exposed (as above) or AECs that had not been UV-exposed (live AECs). As expected live AECs stimulated a significantly attenuated macrophage response compared to UV-exposed AECs (Supplemental Fig. 4).

**Fig. 3:**
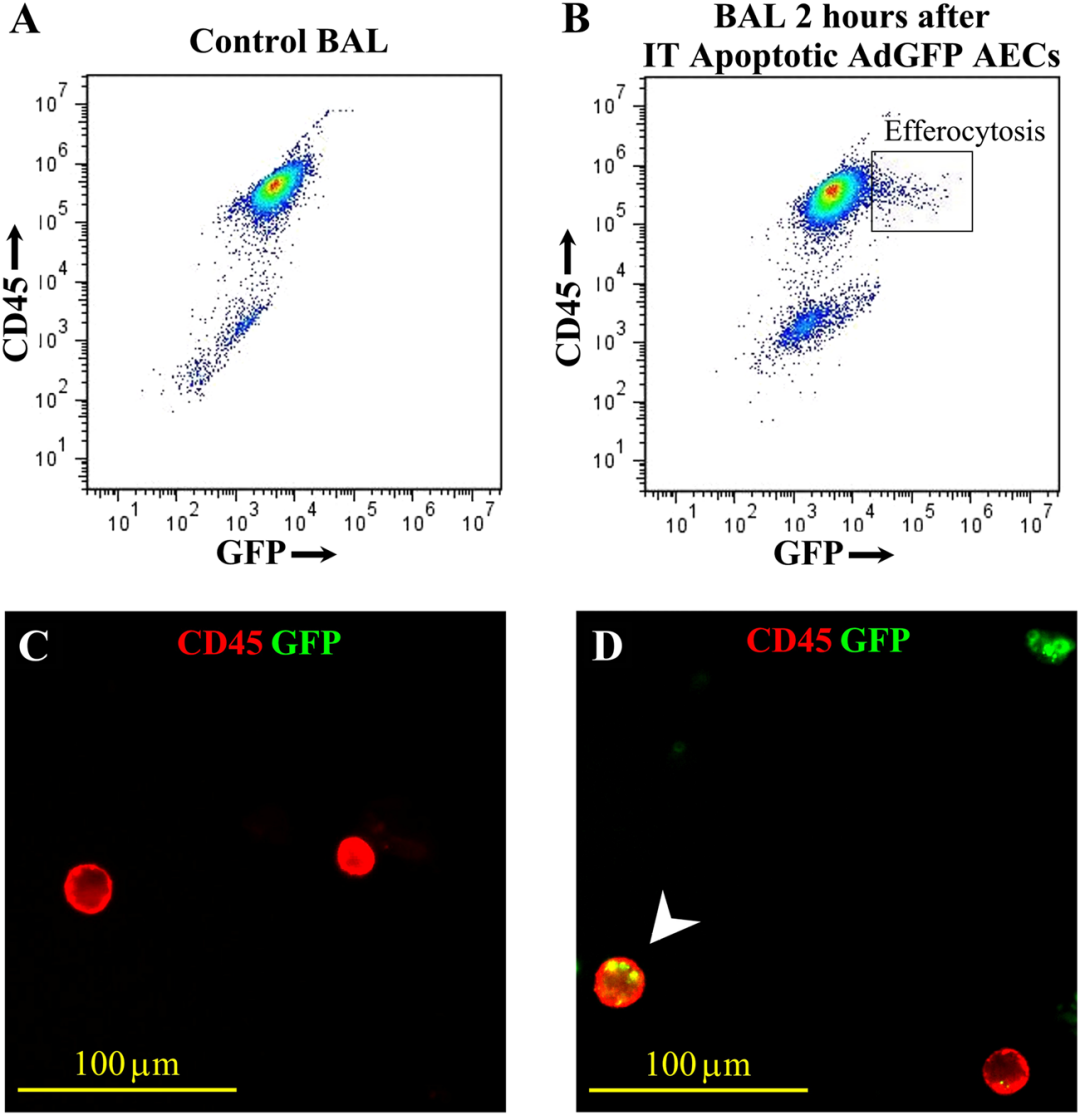
Alveolar macrophages efferocytose apoptotic type II alveolar epithelial cells in vivo. **a**, **b** Flow cytometetry of BAL cells labeled with PE-conjugated anti-mouse CD45 antibody from WT mice 2 h after delivery of PBS only (control) **(a)** or GFP-labeled apoptotic type II alveolar epithelial cells (AEC) **(b)**. **c**, **d** Fluorescent microscopy (100x) of BAL cytospins labeled with PE-conjugated anti-mouse CD45 antibody from WT mice 2 h after delivery of PBS **(c)** or GFP-labeled apoptotic AECs **(d)**. A dual-positive cell stained for CD45 and GFP is demonstrated (arrowhead)

To further assess for in vivo evidence of alveolar macrophage efferocytosis of apoptotic type II AECs, we delivered to uninjured mice by oropharyngeal aspiration a single aliquot of GFP-labeled UV-exposed primary type II AECs. Two hours after the adminsitration, lavage fluid was harvested and BAL cells were analyzed by flow cytometry for GFP and CD45 to broadly identify non-epithelial cells which have taken up GFP-positive apoptotic cells. We identified a population of cells that were positive for both GFP and CD45, providing evidence for engulfment of GFP-labeled apoptotic cells by alveolar macrophages (Fig. 3a, b). Evidence of GFP-labeled apoptotic cells within CD45-positive alveolar macrophages was further confirmed by fluorescent microscopy (Fig. 3c, d).

### Intrapulmonary administration of apoptotic alveolar epithelial cells induced fibrosis

**Fig. 4:**
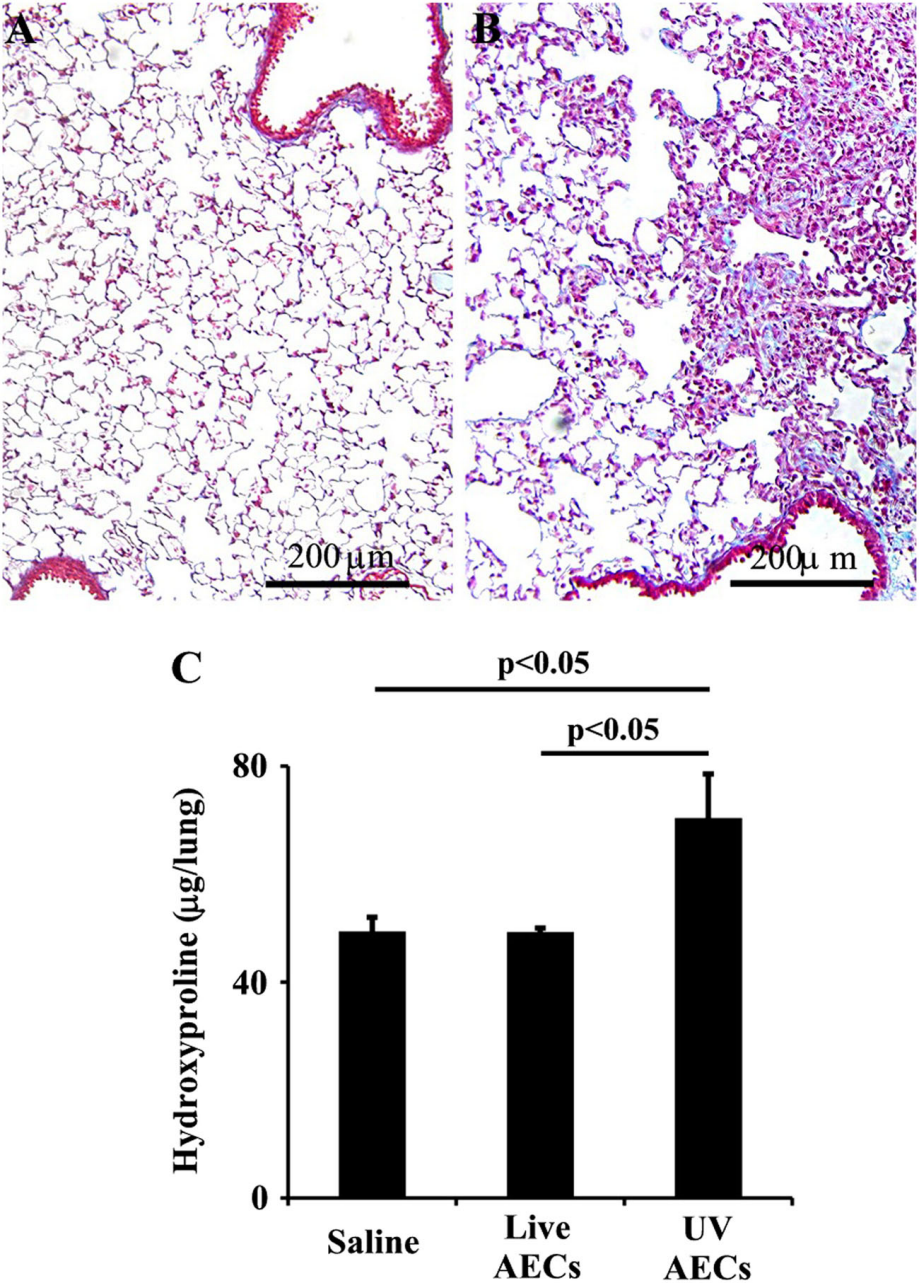
Efferocytosis of UV treated primary alveolar epithelial cells induces fibrosis. **a**, **b** Trichome stained lung sections (20 × ) from mice 21 days after initial delivery of live **(a)** or UV-treated primary alveolar epithelial cells (AECs) **(b)**. **c** Hydroxyproline assay of lungs from mice treated daily doses of PBS, live AECs or UV-treated AECs for 7 days and analyzed 21 days after the initial dose, *n* = 6/group

After we determined that alveolar macrophages ingest apoptotic type II AECs and that this uptake induces a phenotypic change with an up-regulation of TGFβ, we next assessed whether the intrapulmonary administration of apoptotic cells is sufficient to cause pulmonary fibrosis. Previously uninjured WT mice were treated with repetitive doses of PBS, live primary, or UV-exposed primary apoptotic type II AECs by oropharyngeal aspiration. After 21 days, the fibrotic response was assessed by hydroxyproline and lung histology. We found that mice treated with live type II AECs had no induction of fibrosis, while mice treated with apoptotic type II AECs demonstrated significant fibrosis, indicating that the response to apoptotic type II AECs is sufficient to cause a fibrotic response without the need for AEC loss or a second insult (Fig. 4).

**Fig. 5:**
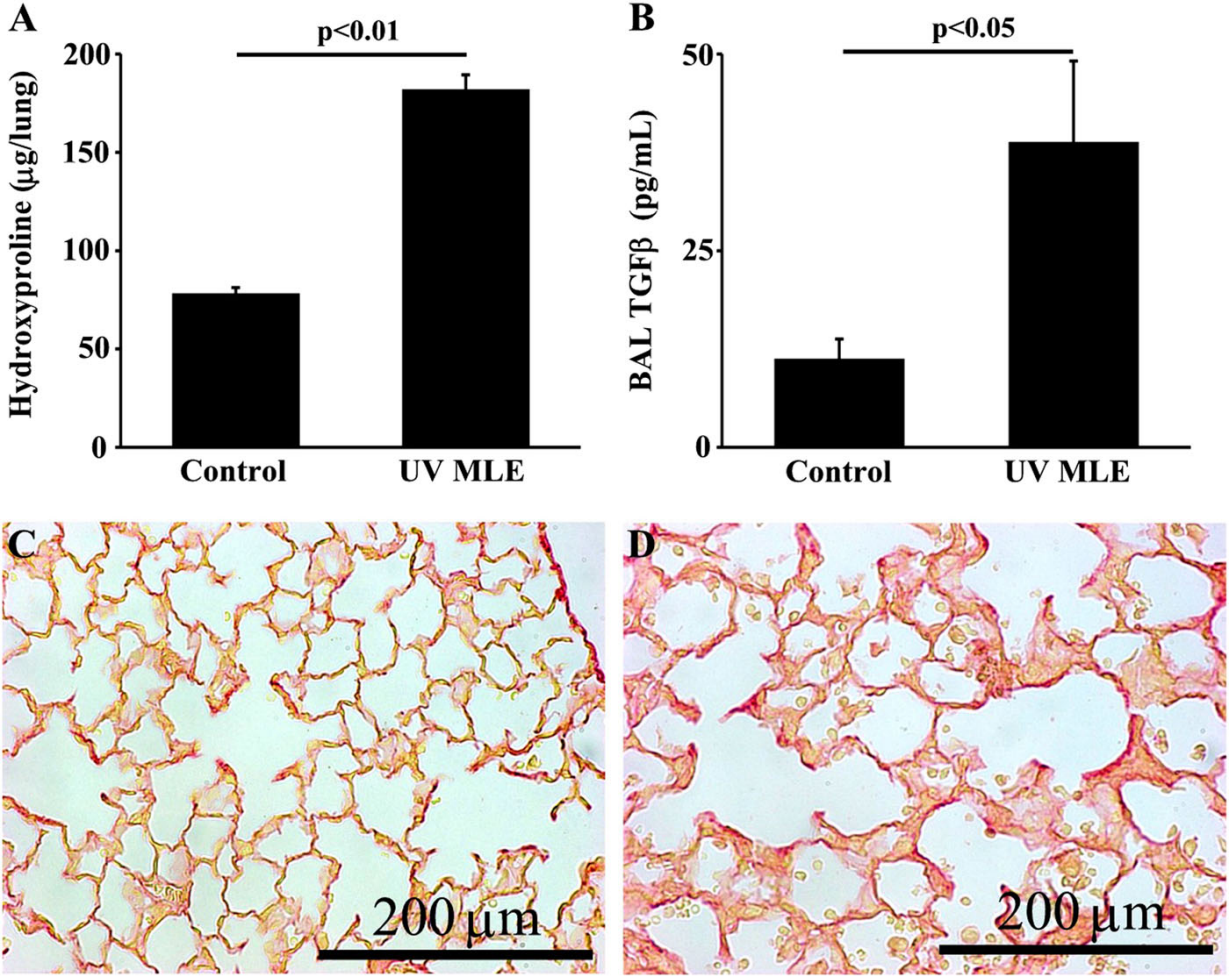
Efferocytosis of UV-treated MLE-12 cells induces fibrosis. **a** Hydroxyproline assay of lungs from mice treated with three times weekly doses of PBS (control) or UV-treated MLE-12 cells for 21 days, *N* = 6/group. **b** TGFβ ELISA of BAL fluid from mice treated with three times weekly doses of PBS or UV-treated MLE-12 cells for 21 days, *N* = 6/group. **c, d** Distribution of fibrosis is shown by picrosiris red stained lung sections (40x) from mice treated with three times weekly doses of PBS **(c)** or UV-treated MLE-12 cells **(d)** for 21 days

To determine whether the observed results were specific to primary type II AECs, we next administered repetitive doses of apoptotic MLE-12 cells, a murine type II AEC line. We confirmed that the administration of apoptotic MLE-12 cells was sufficient to cause significant lung fibrosis. Analysis of lavage fluid from the mice treated with MLE-12 apoptotic cells demonstrates increased concentrations of active TGFβ (Fig. 5). Importantly, we found that repeated administrations of UV-exposed Jurkat cells does not induce a fibrotic response (Supplemental Fig. 3C) consistent with prior reports^22^.

### Apoptotic bodies induced fibrosis is mediated by CD36

**Fig. 6:**
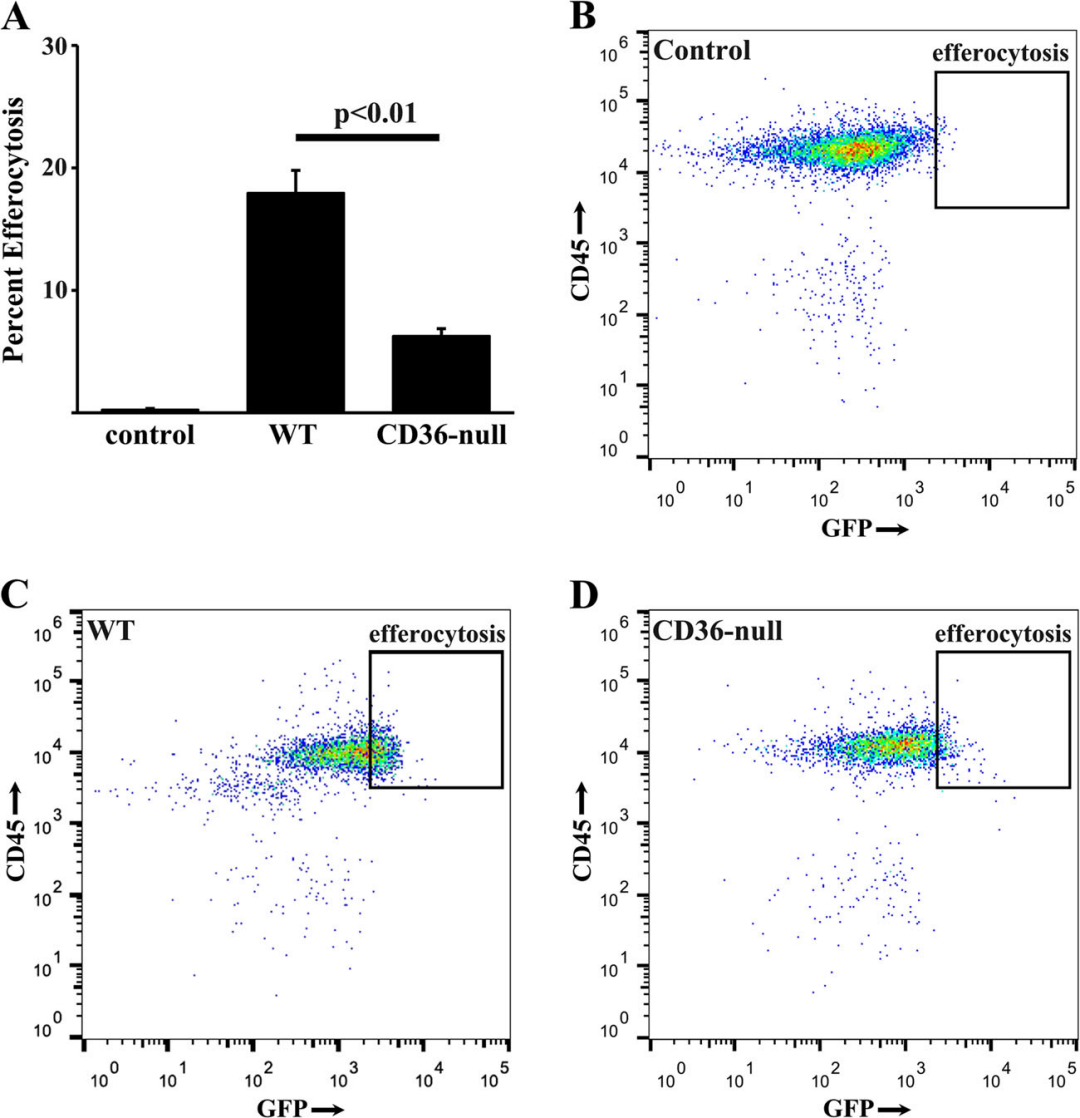
CD36-null mice exhibit less apoptotic type II AEC efferocytosis. **a** BAL cells from WT mice (control) or CD36-null mice 2 h after delivery of PBS or GFP-labeled apoptotic MLE-12 cells were labeded with PE-conjugated anti-mouse CD45 antibody and the percent efferocytosis is quantified by flow cytometry. **b-d** Representative flow cytometry plots of PBS treated WT control mice **(b)**, UV GFP MLE-12 treated WT mice **(c)** and UV GFP MLE-12 treated CD36-null mice **(d)**. *N* = 6 per group

CD36 has been implicated as a key receptor involved in efferocytosis of apoptotic inflammatory cells during resolution of acute lung injury. To determine if CD36 is involved in efferocytosis of apoptotic type II AECs, we delivered UV-treated GFP-labeled MLE-12 cells to WT and CD36-null mice. Two hours after intrapulmonary instillation of the apoptotic bodies, BAL cells were anaylzed for co-expression of CD45 and GFP as an indicator of alveolar macrophage-mediated efferocytosis. While WT cells demonstrated a significant percentage of CD45-positive cells that were GFP-positive, BAL cells from CD36-null mice had much less efferocytosis (Fig. 6).

**Fig. 7:**
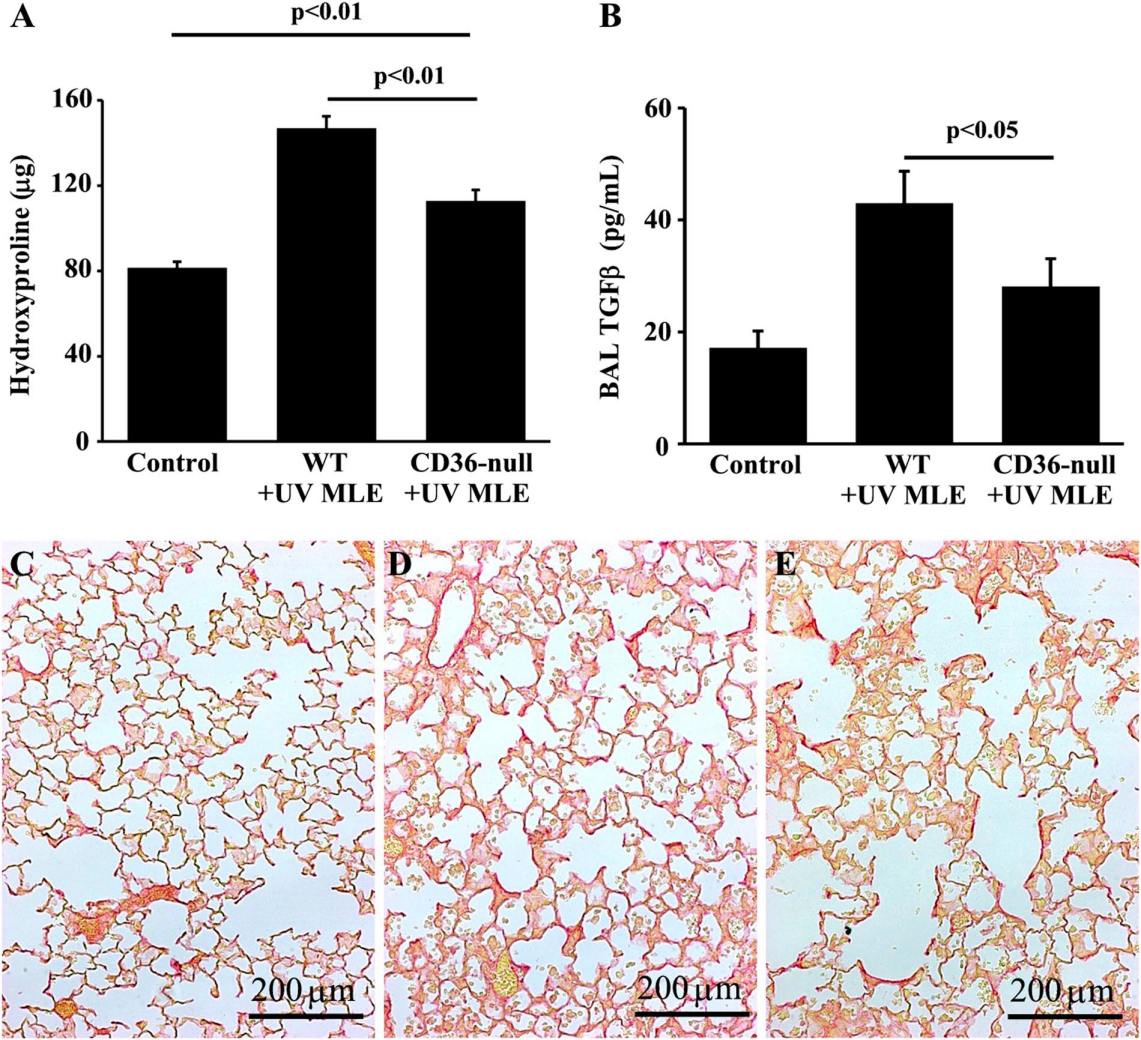
CD36-null mice are protected from efferocytosis-induced fibrosis. Twenty-one days after three times weekly doses of UV-treated MLE-12 cells, CD36-null mice has less fibrosis assessed by hyroxyproline assay **(a)** BAL fluid TGFβ measured by ELISA **(b)** and distribution of fibrosis assessed by histology (20×) of picrosiris red stained lungs slices of PBS treated control mice **(c)**, UV MLE-12 injured WT mice **(d)**, and UV MLE-12 injured CD36-null mice **(e)**. *N* = 6 per group

Finally, to further confirm the critical role of efferocytosis in the induction of fibrosis, we compared the response of WT mice to CD36 null mice following repetitive adminsitrations of apoptotic MLE-12 cells. Consistent with our earlier data, WT mice had a robust fibrotic response to apoptotic MLE-12 cells. While CD36-null mice also exhibited an increase in hydroxyproline, the levels were significantly attenuated compared to WT mice. Similarly, WT mice treated with apoptotic cells for 21 days had significantly higher levels of TGFβ in the BAL fluid compared to CD36-null mice treated with apoptotic cells (Fig. 7). Collectively, these data demonstrate a role for CD36-mediated efferocytosis of apoptotic type II AECs as a driver of fibrosis.

## Discussion

Mounting evidence links defects of the type II AECs with the development of interstitial fibrosis. For example, mutations in type II AEC-specific genes have been identified in familial cases of pulmonary fibrosis^10^. Although the exact mechanism by which these mutations predispose to the disease is unknown, experimental results indicate that the defective protein product induces cell stress which, in turn, predisposes to apoptosis. Indeed, histologic analysis of fibrotic lung tissue highlights the prevalence of AEC apoptosis in areas of ongoing fibrosis. We and others have also shown that induction of apoptosis specifically within AECs is sufficient to initiate a process of fibrosis, which leads to activation and recruitment of multiple different cell types, which ultimately leads to progressive scarring^11,23^. Although it is likely that seveal mechanisms translate the epithelaial abnormalities into a fibrotic repsonse, we demonstrate for the first time that apoptotic AECs are ingested by alveolar macrophages which results in a phenotypic shift that favors profibrotic gene expression. While we cannot exclude the possibility that repetetive admistration of anesthesia and PBS with or without live AECs to the lung induces transient hypoxia and injury, mice exposed to live cells or PBS alone exhibit minimal to no inflammation or fibrosis (Fig. 4a). We therefore demonstrate for the first time that the repetitive administration of apoptotic type II AECs into the lungs is sufficient to drive lung fibrosis independent of a significant secondary insult. Together these findings offer new insight to the downstream events connecting alveolar injury and death to the initiation of fibrogenesis.

Based on histopathologic analyses, denudation of the alveolar epithelium is commonly observed in areas of active fibrosis. Uhal and colleagues identified evidence of increased alveolar epithelial cell apoptosis in tissue sections from patients with fibrotic lung disease, implicating cell death as a mechanism to explain the denuded epithelium overlying the fibrotic lesion^24^. AEC apoptosis has also been identified as a prominent feature in the bleomycin animal model of lung fibrosis^9,20,25^. In our present study, we employed transgenic mice with restricted expression of the diphtheria toxin receptor on their type II AECs. In prior work, we found that repetitive doses of diptheria toxin to target the type II alveolar epithelium induced lung fibrosis^11^. We also discovered that DT treatment did not alter the number of type II AECs in the lung compared to control animals. However, the transgenic mice exhibited increased type II AEC proliferation in order to maintain an equivalent number of cells. This observation suggested to us that DT treatment was causing cell loss by some mechnaism. In the present study, we confirm with a measure of whole lung caspase 3/7 activation that repetitive DT adminsitrations to SPC-DTR-expressing mice causes an increased in apoptosis. This consistent observation of increased AEC apoptosis in human fibrotic lung disease and in distinct animal models certainly implicates this event as a critical element of dsiease pathogenesis. Further evidence substantiating the link between AEC apoptosis and fibrogenesis comes from studies in which fibrosis is ameliorated through interventions that reduce apoptosis of the epithelium^9,26^. Despite the strong correlation between AEC apoptosis and fibrosis, the precise mechanism linking these processes has been unclear.

Apoptosis is a regulated process that can be initiated by various insults or other stimuli^12,27^. Efferocytosis (or the phagocytic uptake of apoptotic cells) relies on surface expression of phophatidylserine (which is normally restricted to the inner plasma membrane leaflet) on the apoptotic cell as a recognition signal for engulfment. Outer leaflet phosphatidylserine expression occurs early in the apoptotic response and persists through the late stages as the cell becomes more and more fragmented, suggesting that macrophages can ingest a broad range of cells and cell debris. In our studies, cells treated with UV light to induce apoptosis were collected after a low speed centrifugation. This protocol likely excluded small bodies from our apoptotic cell preps, but we cannot exclude the possibility that fragments from later stages of apoptosis were included in our in vitro and in vivo experiments. Efferocytosis provides a homeostatic function in tissues by preventing the dead cells from releasing pro-inflammatory molecules and antigens that have the potential to cause autoimmunity^12^. In relation to this homeostatic function, the engulfment of apoptotic cells classically induces an anti-inflammatory or wound healing phenotype in the ingesting cell. For example, previous reports demonstrate that the uptake of apoptotic cells by macrophages results in the increased expression of TGFβ, a growth factor with both anti-inflammatory and pro-fibrotic activities^13^. Based on this mechanistic link between efferocytosis and TGβ production, we hypothesized that persistent alveolar epithelial cell apoptosis, as occurs in lung fibrosis, might lead to protracted TGFβ expression as a result of ongoing ingestion of apoptotic cells by alveolar macrophages and that this macrophage response might translate into fibrosis rather than tissue homeostasis. In deed, we found that repetitive doses of either primary type II AECs or MLE12 cells resulted in lung fibrosis. Our observation that apoptic cell instillation (with ingestion by alveolar macrophages) drives fibrosis is consistent with a report in rats in which a single adminsitration of apoptotic lavaged cells (presumably primarily macrophages) caused lung fibrosis as assessed by sirius red staining of tissue sections^28^. Although we have not assessed the minimum frequency and/or number of apoptotic type II AEC administrations required for the induction of fibrosis in our mouse model, it is remarkable that a single instillation of the apoptotic lavaged cells in rats was sufficient to cause pathology. The authors did find evidence of persistent apoptosis in the rat lungs following the intratracheal delivery of apoptotic cells, and we speculate that this secondary apoptosis may be critical to the promotion of fibrogenesis. Importantly, the authors also detected persistently increased levels of TGFβ in BAL fluid from the rats at the same late time points where they observed evidence of ongoing lung cell apoptosis^12,27^

In contrast to our study results in which the intrapulmonary administration of apoptotic type II AECs elicited a fibrotic response, the intrapulmonary instillation of apoptotic Jurkat cells was found to protect against lung scarring induced by bleomycin injury^22^. In this report, a single administration of apoptotic cells was delivered on day 2 after bleomycin, and the protection against fibrosis was mechanistically linked to an upregulation of hepatocyte growth factor expression that was detectable by day 3 and persisted through day 21. Interestingly, lavage fluid TGFβ levels were suppressed at day 14 and day 21 in the mice that received the apoptoic Jurkat cells, and on day 7, several markers of apoptosis were also decreased. In a follow up study, the apoptotic Jurkat cells were shown to protect against fibrosis by signaling through PPARgamma^29^. Methodological differences between our study and the findings of Lee and Yoon and colleagues likely explain the discrepant results. These methodologic differences include the apoptotic cell type (type II AECs versus Jurkat cells), the number of apoptotic cell administrations (repeated versus a single aliquot), and the presence/absence of an initiating injury to the lung. The funciton of impaired or dysregulated efferocytosis in human disease including IPF is an active area of investigation^12,30^. Notably, alveolar macrophages are not the only cell type which can engulf apoptotic cells^12,27^. Furthermore recruited non-resident macrophages or other inflammatory cell types may also engulf apoptotic cells and contribute significantly to the fibrotic process^31^. Studies of the mechansims involved in regulating expression of efferocytosis receptors and recruitment of different cell types with potentially different responses to apoptotic cell ingestion are currently underway.

The disparate results observed with apoptotic Jurkat cells and type II AECs suggests that the source of apoptotic cells is critical to the down-stream consequences. A number of cell serface receptors have been identified to be important for uptake of leukocyte-derived apoptotic bodies in the context of acute injuries. In contrast, the relevance and importance of specific efferocytosis receptors in the context of AEC-derived apoptotic cells during pulmonary fibrosis has not been studied. In addition to their cell surface receptors for efferocytosis, epithelial cells and leukocytes exhibit distinct functions which could result in differences in the bioactivity of the derived apoptotic cells. Even among epithelial cells, AECs may be fairly unique given their role in generating phospholipid rich surfactant. Recently, Summers and colleagues reported that intrapulmonary delivery of oxidized phospholipids was sufficient to induce pulmonary fibrosis in an animal model^32^. In this same study, it was noted that, in more traditional models of lung fibrosis such as bleomycin injury, there is an accumulation of lipid rich macrophages. This raises the intriguing possibility that AEC apoptosis, which is known to involve activation of ROS, leads to a unique apoptotic body which contains high levels of pro-fibrotic oxidized phospholipids. Notably, CD36 has been identified both as a receptor for efferocytosis as well as a receptor for free phospholipids, and we demonstrate that this receptor is critical to the development of apoptotic type II AEC-induced fibrosis. The importance of CD36 in our model is consistent with findings following bleomycin-induced injury^15^ and other models of fibrosis^33^. Although bleomycin is known to induce AEC apoptosis, in this report we show that the apoptotic cells provoke fibrosis in a CD36-dependent manner in a model in which healthy AECs are preserved at the onset of injury. Although our studies focus on alveolar macrophages, many different cell types are known to engulf apoptotic cells/bodies and likley contribute to the fibrotic response. After efferocytosis, the engulfed particle is rapidly degraded. Therefore, our observation that macrophages from CD36-deficient mice contained fewer apoptotic cell fragments could be explained by mechanisms other than impaired uptake. For example, it is possible that CD36 deficiency simply slows the rate of efferocytosis or accelerates degradation of the engulfed particle. The mechanistic role for CD36 and other efferocytosis receptors in mediating fibrosis through the rate of uptake of apoptotic cells/bodies, the rate of degradation of the engulfed particle, and the regulation of phenotypic responses in different cell types are active areas of investigation in our laboratories.

In summary, we demonstrate that AEC apoptosis promotes fibrosis, in part, through efferocytosis which causes the pro-fibrotic activation of macrophages. Deletion of a key efferocytosis receptor, CD36, significantly blocks efferocytosis-induced fibrosis. Since AEC apoptosis is a prominent feature of fibrotic lung diseases, this AEC apoptosis-efferocytosis-macrophage activation pathway offers novel therapeutic targets.

## Electronic supplementary material

Supplemental Figure Legends

Supplemental Figure 1

Supplemental Figure 2

Supplemental Figure 3

Supplemental Figure 4
