# Supplementary material for: Efferocytosis of apoptotic alveolar epithelial cells is sufficient to initiate lung fibrosis
Source: Cell Death Dis. 2018 Oct 17;9(11):1056. doi: 10.1038/s41419-018-1074-z (PMC6193049; doi:10.1038/s41419-018-1074-z)
Supplement: Supplementary file 1 — Supplemental Figure Legends [file 41419_2018_1074_MOESM1_ESM.docx]

**Supplemental Figures**

**Supplemental Figure 1. Increased TUNEL Staining Following Targeted Type II Alveolar Epithelial Cell Injury.**

WT mice (**A**) or transgenic SPC-DTR mice (**B**) were treated with daily doses of diphtheria toxin (DT, 10 µg/kg i.p.) for 14 days. Lungs were stained for TUNEL (red) and pro-SPC (green). Several TUNEL/pro-SPC co-positive cells are indicated (arrows).

**Supplemental Figure 2**. Surface expression of phosphatidylserine was determine by labeling with FITC-conjugated annexin V and flow cytometry 24 hours after AECs were treated with or without UV light.

**Supplemental Figure 3. Efferocytosis of UV-treated AECs versus UV-treated Jurkat Cells.**

**A.** RT-qPCR expression analysis of cultured primary alveolar macrophages treated with or without UV-treated primary AECs or UV-treated Jurkat cells for 24 hours. **B.** TGFβ ELISA of conditioned media from alveolar macrophages treated with or without UV-treated primary AECs or UV-treated Jurkat cells for 24 hours. N=4, *p<0.05 compared to macrophages stimulated with UV-treated AECs. **C.** Hydroxyproline assay of lungs from mice treated with three times weekly doses of PBS, UV-treated MLE-12 cells, or UV-treated Jurkat cells for 21 days, n=6/group.

**Supplemental Figure 4. Efferocytosis of UV-treated AECs versus Live AECs.**

**A.** RT-qPCR expression analysis of cultured primary alveolar macrophages treated with or without UV-treated primary AECs or uninjured, live primary AECs for 24 hours. **B.** TGFβ ELISA of conditioned media from alveolar macrophages treated with or without UV-treated primary AECs or uninjured, live primary AECs for 24 hours. N=4, *p<0.05 compared to macrophages stimulated with UV-treated AECs.
