# Supplementary figures and images for: Efferocytosis of apoptotic alveolar epithelial cells is sufficient to initiate lung fibrosis
Source: Cell Death Dis. 2018 Oct 17;9(11):1056. doi: 10.1038/s41419-018-1074-z (PMC6193049; doi:10.1038/s41419-018-1074-z)

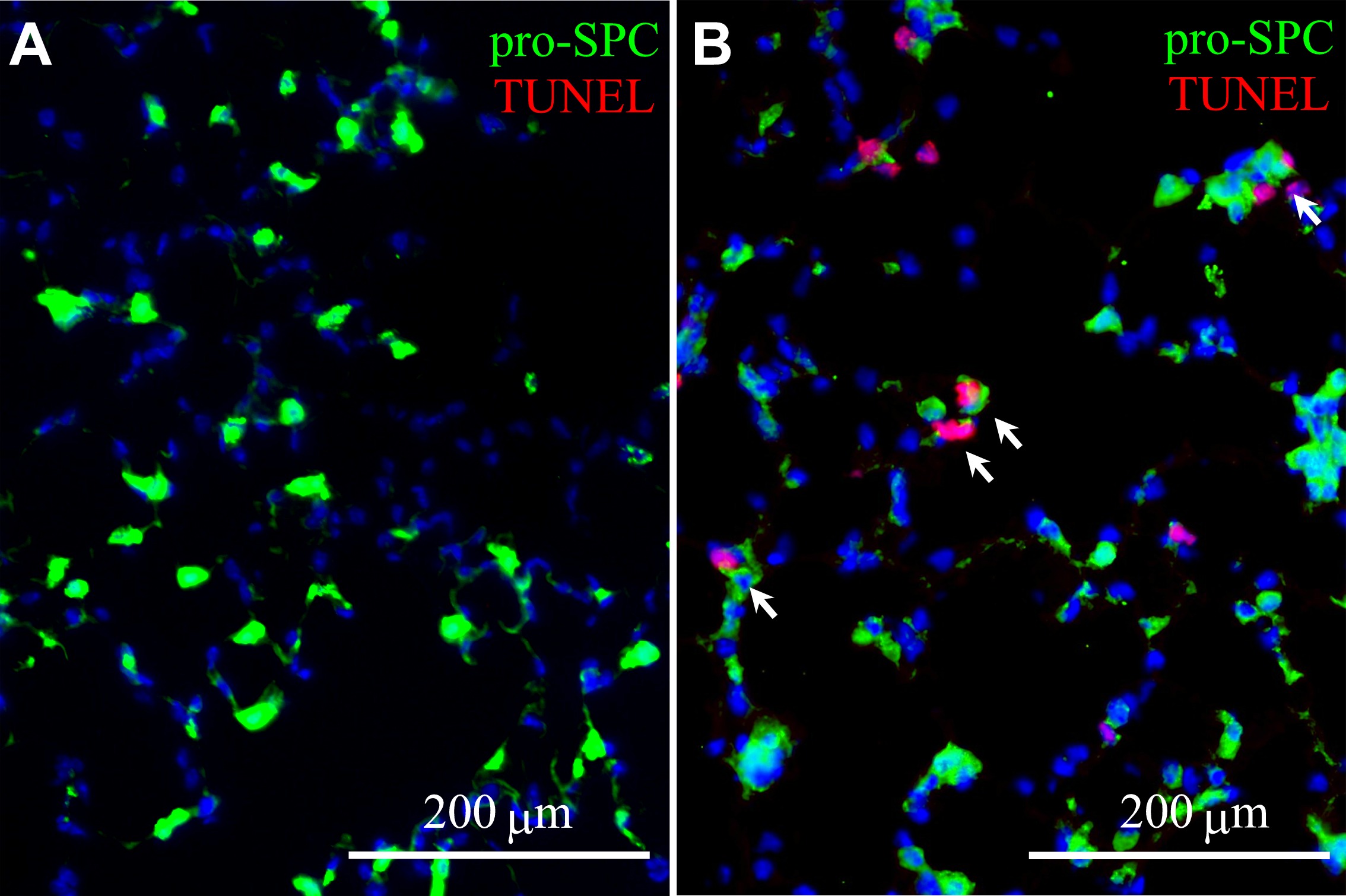

Supplement: Supplementary file 2 — Supplemental Figure 1 [file 41419_2018_1074_MOESM2_ESM.jpg]

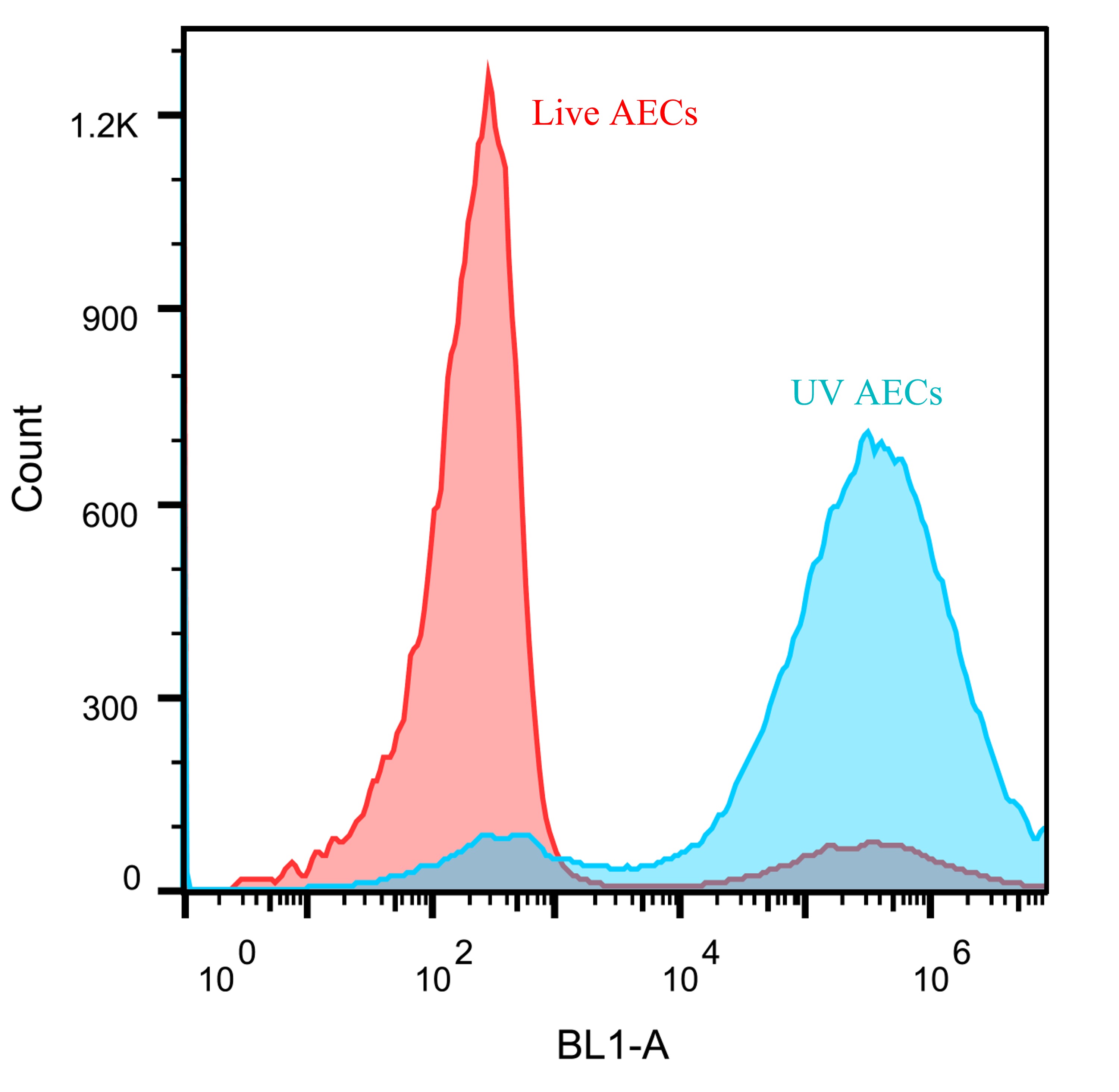

Supplement: Supplementary file 3 — Supplemental Figure 2 [file 41419_2018_1074_MOESM3_ESM.jpg]

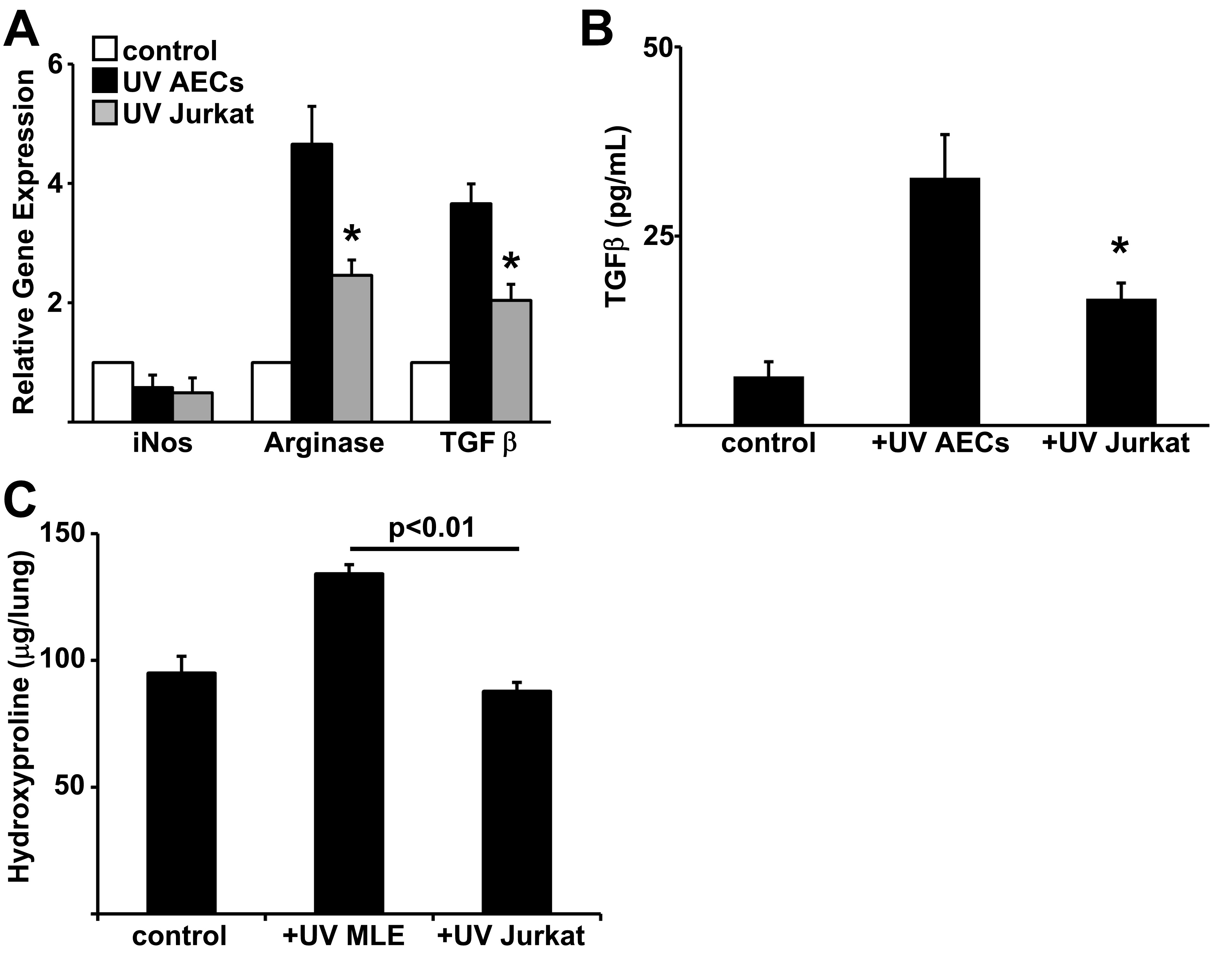

Supplement: Supplementary file 4 — Supplemental Figure 3 [file 41419_2018_1074_MOESM4_ESM.jpg]

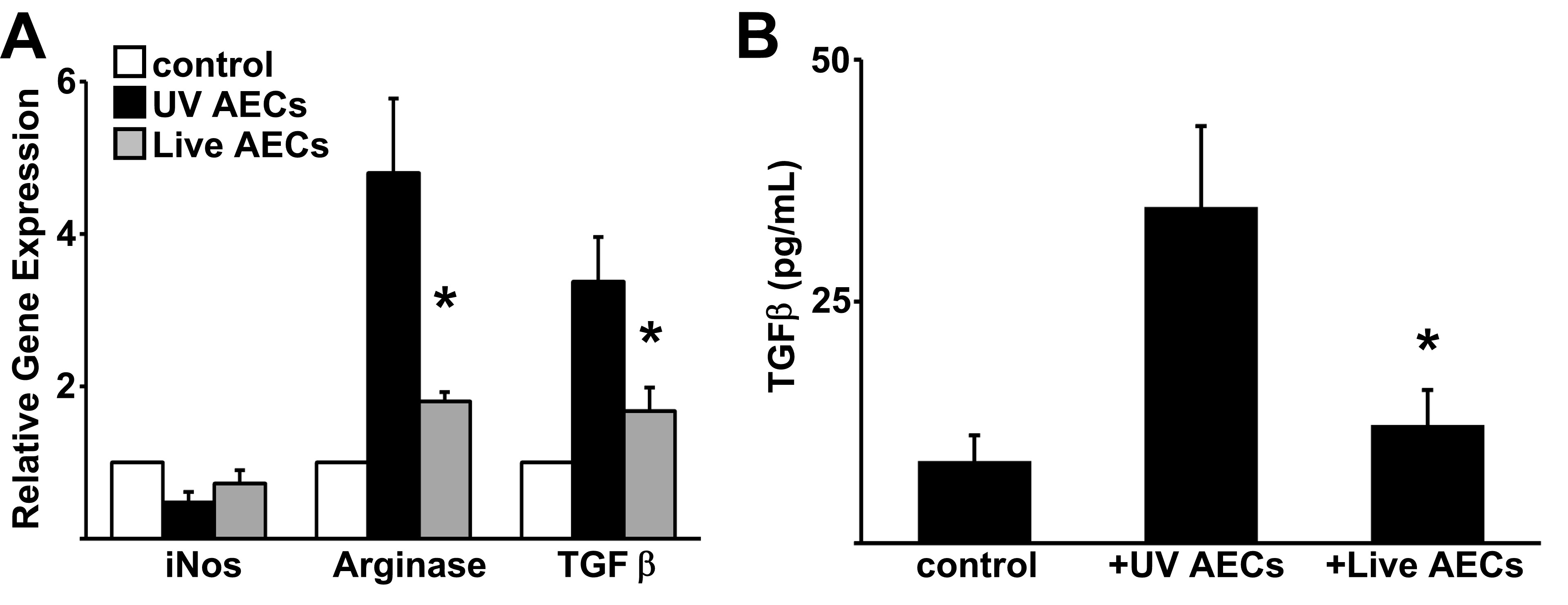

Supplement: Supplementary file 5 — Supplemental Figure 4 [file 41419_2018_1074_MOESM5_ESM.jpg]
